# Cytokine Signatures in Mucocutaneous and Ocular Behçet’s Disease

**DOI:** 10.3389/fimmu.2017.00200

**Published:** 2017-02-27

**Authors:** Giuseppe Lopalco, Orso Maria Lucherini, Antonio Lopalco, Vincenzo Venerito, Claudia Fabiani, Bruno Frediani, Mauro Galeazzi, Giovanni Lapadula, Luca Cantarini, Florenzo Iannone

**Affiliations:** ^1^Department of Emergency and Organ Transplantation, Rheumatology Unit, University of Bari Aldo Moro, Bari, Italy; ^2^Research Center of Systemic Autoinflammatory Diseases and Behçet’s Disease Clinic, Department of Medical Sciences, Surgery and Neurosciences, University of Siena, Siena, Italy; ^3^Department of Pharmaceutical Chemistry, University of Bari, Bari, Italy; ^4^Department of Ophthalmology, Humanitas Research Hospital, Milan, Italy

**Keywords:** Behçet’s disease, cytokines, mucocutaneous involvement, ocular disease, signature

## Abstract

Behçet’s disease (BD) is a multi-systemic inflammatory disorder consisting of recurrent oral aphthosis, genital ulcers, and chronic relapsing bilateral uveitis; however, many other organs may be affected. Several pro-inflammatory cytokines, mainly derived from Th1 and Th17 lymphocytes, seem to be involved in different pathogenic pathways leading to development of the clinical manifestations. On this basis, the primary aim of our study was to compare a core set of pro-inflammatory cytokines between patients with BD and healthy control (HC). The secondary goal was to evaluate potential correlations between these putative circulating biomarkers, the status of disease activity, and the specific organ involvement at the time of sample collection. Fifty-four serum samples were collected from 46 BD patients (17 males, 29 females, mean age 45.5 ± 11.3 years), and 19 HC (10 males, 9 females, mean age 43 ± 8.3 years). Twenty-five serum cytokines (APRIL/TNFS13, BAFF/TNFSF13B, sCD30/TNFRSF8, sCD163, Chitinase3-like1, gp130/sIL-6Rb, IFNb, sIL-6Ra, IL-10, IL-11, IL-19, IL-20, IL-26, IL-27 (p28), IL-28A/IFN-lambda2, IL-29/IFN-lambda1, IL-32, IL-34, IL-35, LIGHT/TNFSF-14, Pentraxin-3, sTNF-R1, sTNF-R2, TSLP, and TWEAK/TNFSF-12) were simultaneously quantified using a Bio-Rad cytokine bead arrays. Serum concentration of sTNF-R1 (*p* < 0.01) and sTNF-R2 (*p* < 0.01) resulted higher in both active and inactive BD than HC, while Chitinase3-like1 (*p* < 0.05) and gp130/sIL-6Rb (*p* < 0.01) serum levels were significantly higher in inactive BD, and IL-26 (*p* < 0.01) in active BD than HC. No differences were observed between inactive and active BD group. In addition, we observed that gp130/sIL-6Rb, sIL-6Ra, IL-35, and TSLP serum levels were significantly enhanced in patients with mucocutaneous manifestations plus ocular involvement (MO-BD) compared to subgroup with only mucocutaneous involvement (M-BD). Our findings may suggest a signature of IL-6, tumor necrosis factor-α as well as of Th17 response in BD patients due to increased levels of gp130/sIL-6Rb, sTNF-R1, sTNF-R2, IL-26, respectively. This evidence could contribute to improve the knowledge regarding the role of these citokines in the induction of specific BD clinical features.

## Introduction

Behçet’s disease (BD) is a rare multisystemic inflammatory disorder clinically characterized by the “triple symptom complex,” consisting of recurrent oral aphthosis, genital ulcers, and relapsing bilateral uveitis. Besides this classical clinical pattern, also other organ engagements including gastrointestinal tract, musculoskeletal, cardiovascular, and central nervous system, are documented (1–3). The recent understandings on cellular and molecular biology seem to suggest that an abnormal activation of both innate and adaptive immunity would be able to generate an inflammatory process leading to a CD4+ T lymphocytes clonal expansion which in turn produces high concentrations of both pro-inflammatory cytokines and cytotoxic CD8+ cells (4–11). The link between innate and adaptive immunity in patients with BD has been further clarified demonstrating that T cell immune response is skewed toward Th1 and Th17 polarization with decreased activity of regulatory T cells (12–14). The Th17 cells, play a critical role in the pathogenesis of a variety of autoimmune inflammatory diseases leading to production of Th17 effector cytokines, namely IL-17, IL-22, and IL-26 (15). This latter cytokine, belonging to the IL-10 family proteins, acts on monocytes to produce other pro-inflammatory mediators, such as IL-1β, IL-6, and tumor necrosis factor (TNF)-α which enhance the generation of new Th17 cells (16). Although the contribution of IL-26 to the development of autoimmune diseases is undoubted, its role in BD pathogenesis is still unclear. Additional cytokines involved in mechanisms known to play a critical role in BD pathogenesis were recently associated to disease activity. Indeed, molecules such as Chitinase3-like1 regulating monocytes differentiation, as well as antibacterial and type 17 responses, was observed to be upregulated in BD patients compared to healthy control (HC). Interestingly, this cytokine was also seen to be associated to disease activity in BD, correlating positively with elevated IL-6 serum levels (17–19).

Tumor necrosis factor-α appears to be crucial in promoting the development of the disease. This is documented by an overproduction of soluble TNF-α receptors and TNF-α sera levels spontaneously secreted by monocytes in active BD patients (20). Yet, elevated levels of TNF-α and soluble tumor necrosis factor receptors sTNFR1 and sTNFR2 have been found in BD patients. Moreover, increased systemic and synovial levels of sTNFRs in active BD strongly suggest a central role for the TNF/TNFR pathway in the pathogenesis of skin and joint involvement (21). Even though it is well documented that TNF blockade reduces the expression of different biological mediators and their receptors, the knowledge in BD regarding the role of the receptor gp130/sIL-6Rb is still poor. This shared receptor is utilized by several related cytokines, including IL-6, IL-11, and IL-27, which in turn regulate cellular recruitment to local sites of inflammation, induce differentiation factor for Th17 cells, promote Th2 differentiation, and inhibit multiple T cell subsets (22).

Upregulation of TNF family members involved in T and B lymphocytes activation including APRIL/TNFSF13, BAFF/TNFSF13B, sCD30/TNFRSF8 were also observed in BD patients underling a potential role of these cytokines in the immune response (23, 24).

Although literature data have proven that several cytokines are involved in BD pathogenesis, to date, biologic markers correlating with the disease activity have not yet been well recognized. Moreover, in the context of the same disease, it is difficult to identify a subset of disease signed by a specific cytokine profile. Therefore, the purpose of this work was to investigate the potential role of specific circulating biomarkers of inflammation involved in adaptive and innate immune response in BD, in order to correlate their circulating levels with clinical manifestations and disease activity.

## Patients and Methods

### Patients

Fifty-four serum samples were routinely collected and analyzed from 46 consecutive BD patients (17 males, 29 females, mean age 45.5 ± 11.3 years), who met the International Study Group Criteria (ISGC) (25) and the International Criteria for BD (ICBD) (26) and from 19 HC (10 males, 9 females, mean age 43 ± 8.3 years) who attending the outpatient clinic at the Rheumatology Unit of the University of Bari and who are negative for BD criteria (ISGC and ICBD). These subjects underwent detailed clinical and laboratory workup, in order to rule out any inflammatory, metabolic, and neoplastic disorders (in particular, they all showed inflammatory markers within normal values). All patients and controls were Caucasians of Italian origin. For seven patients, more than one serum sample was obtained during an active phase of disease, resulting in a total of 54 BD samples. Moreover, the samples were collected every 3 to 4 months and in case of disease relapse. Table 1 summarizes the clinical and demographic characteristics of BD patients. The primary aim of the study was to compare a cytokine profile between BD patients and HC; the secondary aim was to evaluate potential correlations between these putative circulating biomarkers, the status of disease activity, and the specific organ involvement at the time of sample collection. According to several other studies correlating circulating biomarkers with disease activity, BD patients were included in active BD group when they had at least two of the following clinical findings: uveitis, oral aphthosis, genital aphthosis, cutaneous disease, and gastrointestinal involvement (6, 9, 27–29). More specifically, anterior and posterior uveitis were observed in 4/13 and 9/13 patients, whereas gastrointestinal involvement was endoscopically characterized by the presence of typical oval ulcers mostly localized in the terminal ileum. Written informed consent was obtained both from patients and HC. The study protocol was reviewed and approved by the Ethical Committee of the Medical University of Bari. Demographic and clinical information was obtained through structured interview, review of medical records, physical examination, and laboratory tests.

**Table 1:** **Demographic, laboratory, and clinical characteristics of patients affected by Behçet’s disease (BD) recruited in our study**.

	BD patients (*n* = 46)	HLA-B51-positive patients (*n* = 24)
Males, *n* (%)	17 (37)	8 (33)
Disease onset (mean ± SD) in years	32.16 ± 10.56	33.47 ± 11.17
Disease duration (mean ± SD) in months	144.5 ± 91.83	106.9 ± 88.49
Patients fulfilled the International Study Group Criteria in %	100	100
Patients fulfilled the International Criteria for BD in %	100	100
Clinical features (%)		
Uveitis	13/46 (28)	8/24 (33)
Oral aphthosis	29/46 (63)	15/24 (62)
Genital aphthosis	7/46 (15)	4/24 (17)
Cutaneous disease	24/46 (52)	12/24 (50)
Gastrointestinal involvement	10/46 (22)	5/24 (21)

### Multiplex Bead Analysis

A panel of 25 serum cytokines [APRIL/TNFS13, BAFF/TNFSF13B, sCD30/TNFRSF8, sCD163, Chitinase3-like1, gp130/sIL-6Rb, IFNb, sIL-6Ra, IL-10, IL-11, IL-19, IL-20, IL-26, IL-27 (p28), IL-28A/IFN-lambda2, IL-29/IFN-lambda1, IL-32, IL-34, IL-35, LIGHT/TNFSF-14, Pentraxin-3, sTNF-R1, sTNF-R2, TSLP, TWEAK/TNFSF-12] were simultaneously quantified using a Bio-Rad cytokine bead arrays according to the manufacturers’ instructions. Data analysis was performed using the Bioplex manager software 6.0.

### Statistical Analysis

Statistical analyses were performed using GraphPad Prism 5 software. Two-tailed Mann–Whitney test (for two non-parametric groups) and Student’s *t*-test (for two parametric groups) were used for statistical comparisons between groups. Significance in multiple comparisons was by one-way analysis of variance with a Bonferroni correction or Kruskall–Wallis test with a Dunn’s multiple comparison correction. Correlations were calculated using Spearman’s correlation (two-tailed *p*-value) as well as Pearson’s correlation test when required. Significance was defined as *p* < 0.05.

## Results

### Clinical Characteristics of BD Patients

Overall, 54 serum samples were obtained from 46 BD patients, and 31 of these (57%) were collected from patients with active disease. The main demographic and clinical characteristics of the subjects involved in this study are shown in Table 1. Moreover, 27 serum samples were obtained from HLA-B51-positive BD patients (50%) and at the time of serum collection patients were receiving the following treatments: TNF inhibitors 21/54, DMARDs combined with corticosteroids 14/54, DMARDs alone 10/54, corticosteroids 3/54, anti-IL-1 agents 3/54, and three patients were no treated.

### Elevated Cytokine Levels of Chitinase3-like1, gp130/sIL-6Rb, IL-11, IL-26, sTNF-R1, and sTNF-R2 in BD Patients in Comparison to Healthy Controls

**Figure 1:**
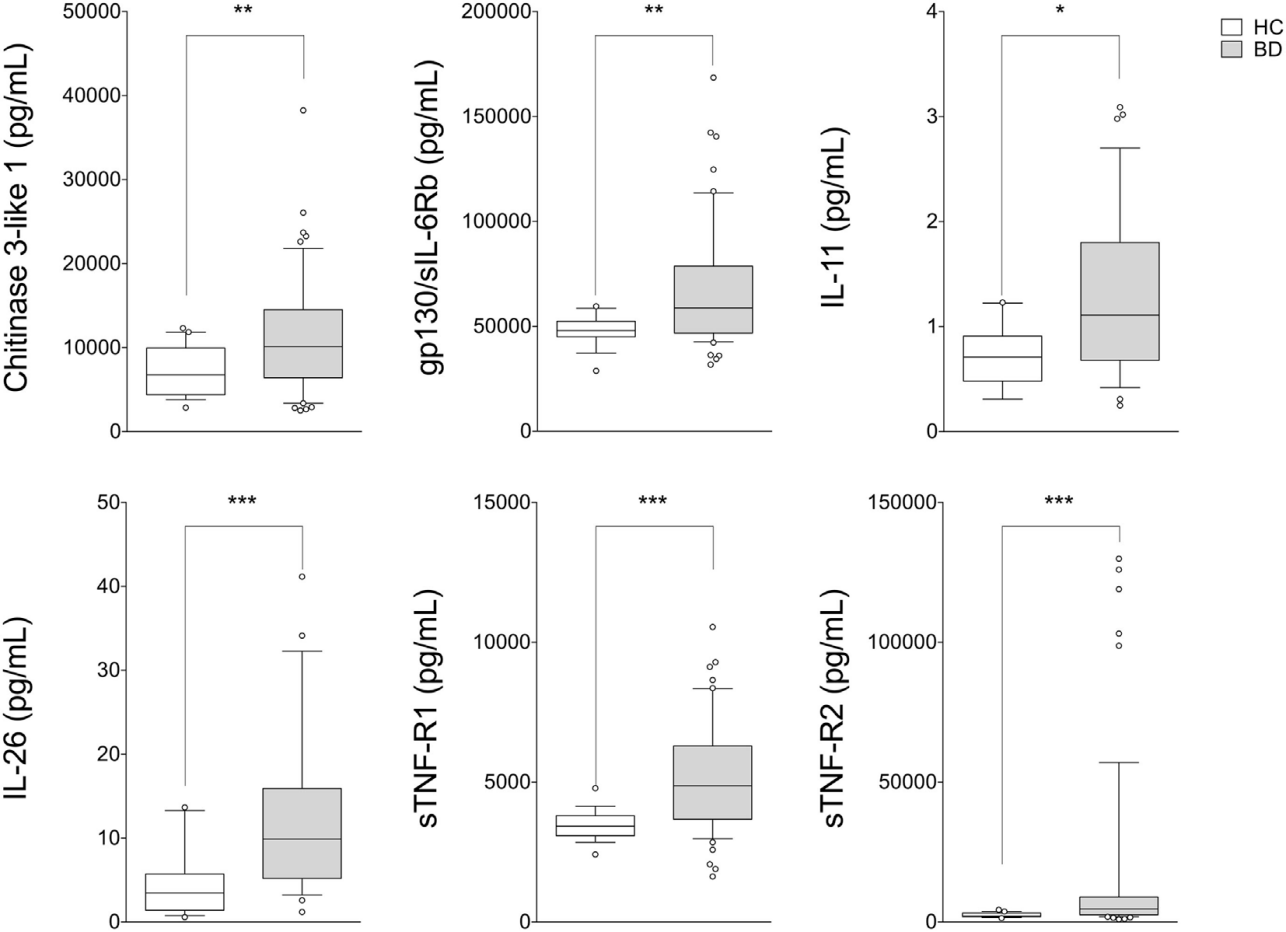
**Serum cytokine profile in patients with BD**. BD patients (*n* = 54) showed upregulation of serum levels of Chitinase3-like1, gp130/sIL-6Rb, IL-11, IL-26, sTNF-R1, and sTNF-R2 compared with HC (*n* = 19). Mann–Whitney *U*-test and Student’s *t*-test were carried out to check for statistical significance between groups when required (***p < 0.001, **p < 0.01, *p < 0.05). The central line represents the distribution median, boxes span 25th to 75th percentiles, and error bars extend from 10th to 90th percentiles. Dots (°) are outlier values, higher than the 90th percentile. Abbreviations: HC, healthy controls; BD, Behçet’s disease.

Circulating levels of 25 cytokines were measured in serum samples obtained from BD patients (*n* = 54) and HC (*n* = 19). Cytokine levels of IL-10, IL-27 (p28), IL-28A/IFN-lambda2, IL-29/IFN-lambda1, IL-32, IL-34, and LIGHT/TNFSF-14 were found in less than 50% of samples collected; for this reason these cytokines were not included in the analysis. No differences between BD patients and HC, in serum levels of APRIL/TNFS13, BAFF/TNFSF13B, sCD30/TNFRSF8, sCD163, IFNb, sIL-6Ra, IL-19, IL-20, IL-35, Pentraxin-3, TSLP, and TWEAK/TNFSF-12 were observed. In contrast, serum levels of Chitinase3-like1 (*p* = 0.009), gp130/sIL-6Rb (*p* = 0.002), IL-11 (*p* = 0.008), IL-26 (*p* < 0.001), sTNF-R1 (*p* < 0.001), and sTNF-R2 (*p* < 0.001) were significantly higher in BD than HC (Figure 1). Correlation study revealed significant correlation between cytokines showed to be upregulated in serum from BD patients. Among these, strong correlation was found between gp130/sIL-6Rb and sTNF-R1 (*r* = 0.706, *p* < 0.001), sTNF-R2 (*r* = 0.783, *p* < 0.001), sCD163 (*r* = 0.775, *p* < 0.001), TWEAK/TNFSF-12 (*r* = 0.775, *p* < 0.001), and sIL-6Ra (*r* = 0.705, *p* < 0.001) serum levels. sTNF-R1 also correlated with TSLP (*r* = 0.730, *p* < 0.001) and sTNF-R2 (*r* = 0.739, *p* < 0.001). Moreover, sTNF-R2 serum levels positively correlated with TSLP (*r* = 0.772, *p* < 0.001) and sCD163 (*r* = 0.724, *p* < 0.001) (Table S1 in Supplementary Material).

### Serum Cytokine Profiles and Their Correlation with Disease Activity and Clinical Features

**Figure 2:**
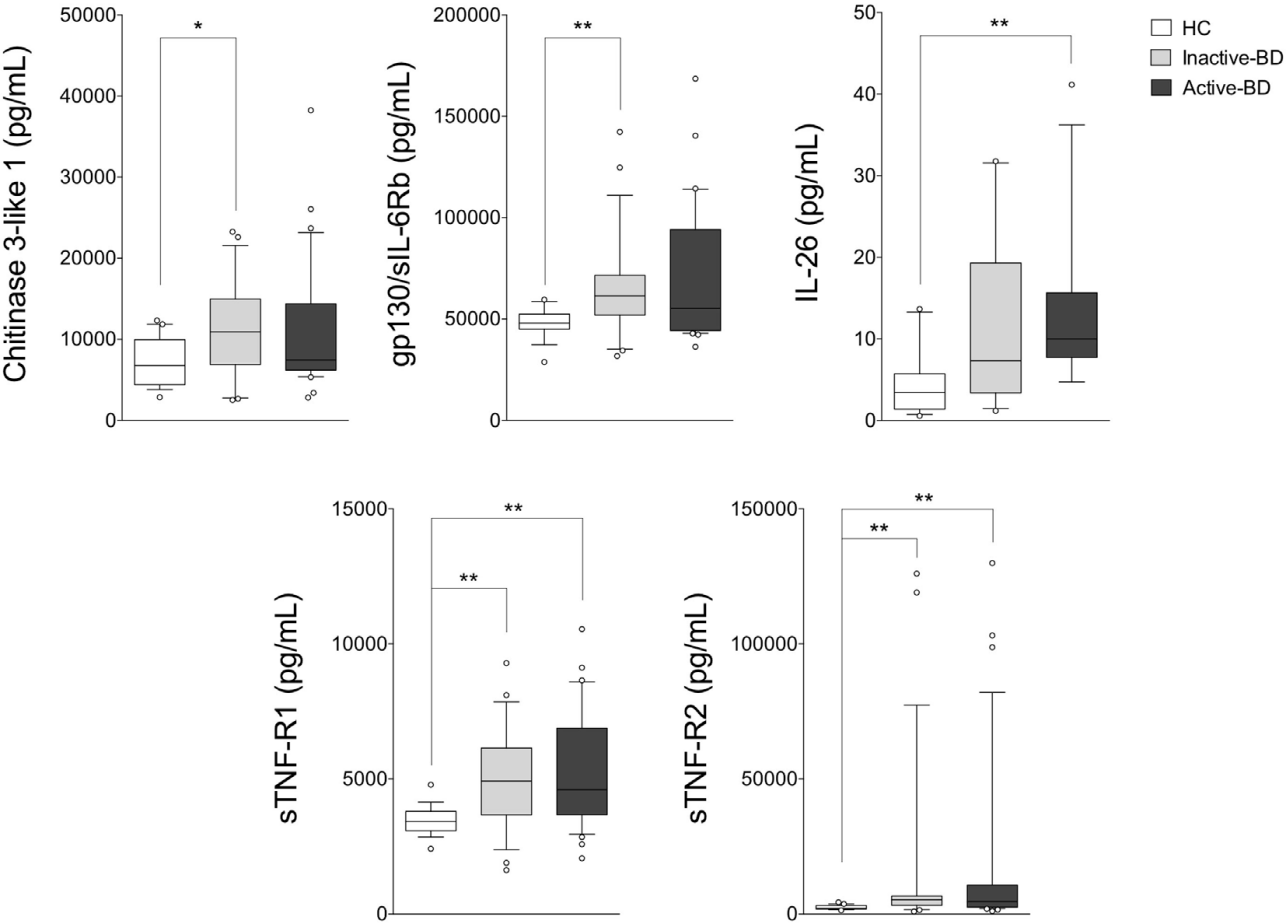
**Serum cytokine profile analysis in BD patients with different disease activity**. Serum cytokine levels in inactive BD (*n* = 23), active BD (*n* = 31) patients, and HC (*n* = 19). Analysis of variance was used for data comparison (***p < 0.001, **p < 0.01, *p < 0.05). The central line represents the distribution median, boxes span 25th to 75th percentiles, and error bars extend from 10th to 90th percentiles. Dots (°) are outlier values, higher than the 90th percentile. Abbreviations: BD, Behçet’s disease; HC, healthy controls.

**Figure 3:**
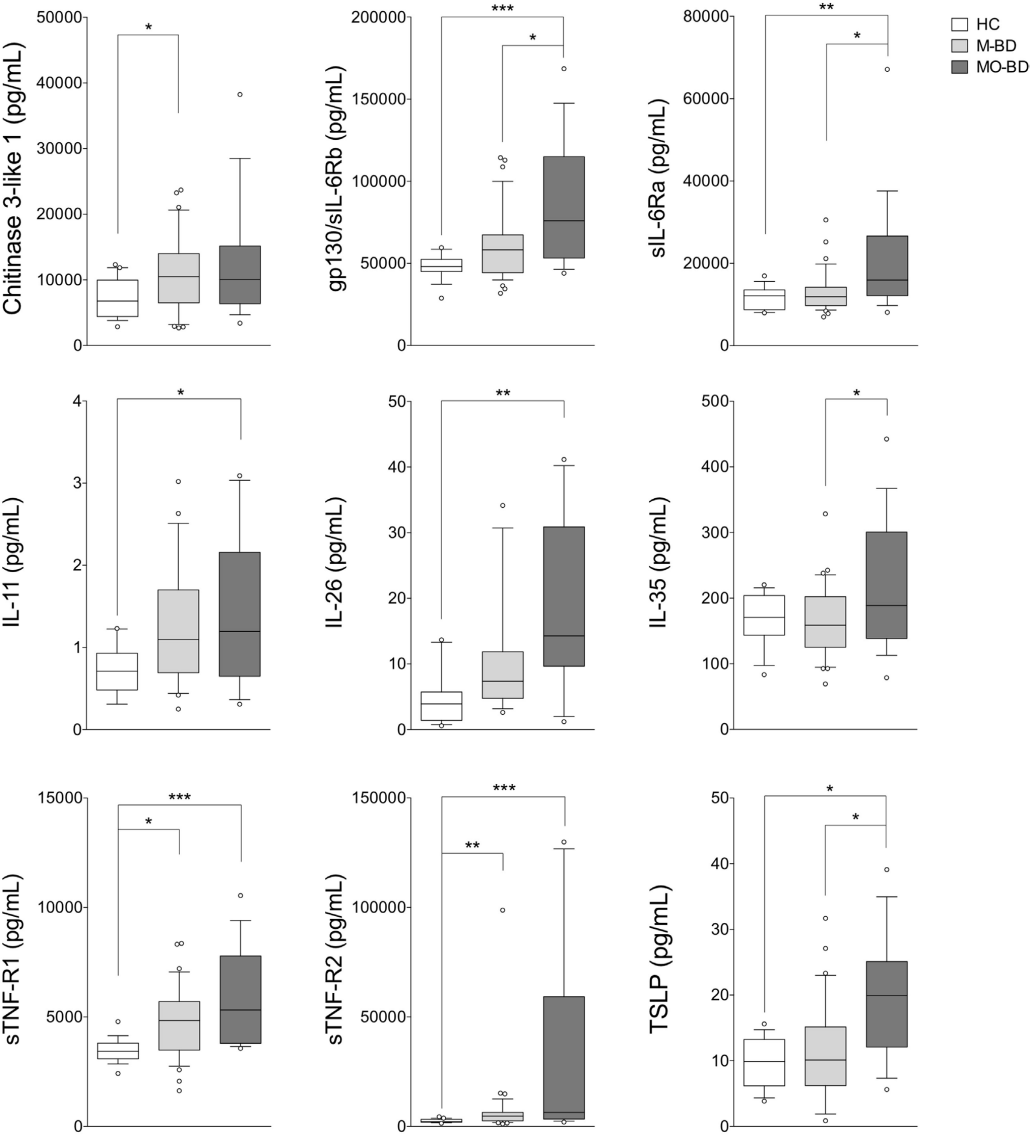
**Serum cytokine profile in mucocutaneous patients with (MO-BD) or without (M-BD) ocular involvement**. Serum cytokine levels in M-BD (*n* = 35), MO-BD (*n* = 17) patients, and HC (*n* = 19). Analysis of variance was used for data comparison (***p < 0.001, **p < 0.01, *p < 0.05). The central line represents the distribution median, boxes span 25th to 75th percentiles, and error bars extend from 10th to 90th percentiles. Dots (°) are outlier values, higher than the 90th percentile. Abbreviations: HC, healthy controls; BD, Behçet’s disease; M-BD, mucocutaneous patients without ocular involvement; MO-BD, mucocutaneous patients with ocular involvement.

As shown in Figure 2, BD patients were divided into two subgroups based on the disease activity. In active BD group were included patients characterized by at least two of the following clinical findings: uveitis, oral aphthosis, genital aphthosis, cutaneous disease, gastrointestinal involvement. Twenty-three serum samples were collected from inactive BD patients (8 males, 15 females, mean age 44.6 ± 11.01 years) and 31 from active patients (12 males, 19 females, mean age 46.1 ± 11.67 years). Serum levels of sTNF-R1 and sTNF-R2 resulted higher in both active BD (*p* = 0.002 and *p* = 0.002, respectively) and inactive BD (*p* = 0.0101 and *p* = 0.002, respectively) subgroup than HC, while Chitinase3-like1 (*p* = 0.042) and gp130/sIL-6Rb (*p* = 0.008) serum levels were significantly higher in inactive BD as well as IL-26 (*p* = 0.002) in active BD than HC (Figure 2). Interestingly, among significantly serum cytokines upregulated in inactive BD and active BD patients, gp130/sIL-6Rb strongly correlated with sTNF-R2 (*r* = 0.716, *p* < 0.001), TWEAK/TNFSF-12 (*r* = 0.821, *p* < 0.001), IL-26 (*r* = 0.773, *p* = 0.007) serum levels, and sTNF-R1 was to correlate with sTNF-R2 (*r* = 0.877, *p* < 0.001), IL-26 (*r* = 0.773, *p* = 0.007), and IL-20 (*r* = 0.709, *p* = 0.0018) serum levels. In contrast, active BD subgroup revealed sTNF-R1 and TSLP (*r* = 0.769, *p* < 0.001) serum levels strong positively correlation. Moreover, sTNF-R2 serum levels positively correlated with TSLP (*r* = 0.830, *p* < 0.001), gp130/sIL-6Rb (*r* = 0.818, *p* < 0.001), and sCD163 (*r* = 0.725, *p* < 0.001) (Table S2 in Supplementary Material). To determine the relationship between clinical features and the circulating levels of inflammatory markers, we measured and compared cytokine levels in serum from patients who presented at the time of blood collection mucocutaneous manifestations with (MO-BD) or without (M-BD) ocular involvement (Figure 3). Results showed significant enhanced levels of Chitinase3-like1 (*p* = 0.039) in M-BD but no differences were identified in MO-BD subgroup (*p* = 0.1035) compared with HC. Serum levels of sTNF-R1 and sTNF-R2 were higher in both M-BD (*p* = 0.0155 and *p* = 0.0066, respectively) and MO-BD (*p* < 0.001 and *p* < 0.001, respectively) than HC. In addition, elevated serum levels of gp130/sIL-6Rb (*p* < 0.001), sIL-6Ra (*p* = 0.005), IL-11 (*p* = 0.027), IL-26 (*p* = 0.002), and TSLP (*p* = 0.0103) were also observed in MO-BD compared to HC. Interestingly, we observed that gp130/sIL-6Rb (*p* = 0.043), sIL-6Ra (*p* = 0.029), IL-35 (*p* = 0.026), and TSLP (*p* = 0.013) serum levels were significantly enhanced in MO-BD compared to M-BD subgroup (Figure 3). Correlation analysis among all evaluated cytokines was also assessed. Notably, stronger significant correlation was revealed in MO-BD compared with M-BD subgroup (Table S3 in Supplementary Material).

### Linear Regression Analysis between Serum Cytokine Levels and Disease Duration

**Figure 4:**
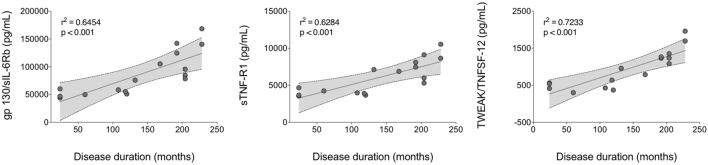
**Linear regression analysis of serum cytokine levels versus disease duration**. A positive strong correlation was found between gp130/sIL-6Rb, sTNF-R1, and TWEAK/TNFSF-12 circulating levels and disease duration in MO-BD (mucocutaneous patients with ocular involvement) subgroup.

A linear regression analysis of cytokine serum levels in all patients as well as in disease activity and clinical features subgroups as a function of disease duration was assessed. No significant correlations were obtained in all patients and in active BD subgroup (Table S4 in Supplementary Material). In contrast, Chitinase3-like1 was found significant in inactive BD, M-BD, and MO-BD subgroup (*r*_s_ = 0.277 *p* = 0.025, *r*_s_ = 0.188 *p* = 0.011, *r*_s_ = 0.274 *p* = 0.038, respectively) (Table S4 in Supplementary Material). Moreover, significant results were found in both M-BD and MO-BD subgroups for sCD163 (*r*_s_ = 0.124 *p* = 0.041 and *r*_s_ = 0.473 *p* = 0.003, respectively) and gp130/sIL-6Rb (*r*_s_ = 0.162 *p* = 0.018 and *r*_s_ = 0.645 *p* < 0.001, respectively). In addition, sIL-6Ra revealed significant correlation in M-BD (*r*_s_ = 0.157 *p* = 0.02) while sTNF-R1 (*r*_s_ = 0.628 *p* < 0.001), TWEAK/TNFSF-12 (*r*_s_ = 0.723 *p* < 0.001), and sCD30/TNFRSF8 (*r*_s_ = 0.39 *p* = 0.010) were significantly correlated to disease duration in MO-BD subgroup (Table S4 in Supplementary Material; Figure 4).

## Discussion

Our study was aimed at investigating a core set of cytokines in a cohort of patients mainly affected by the most common clinical manifestations of BD, including mucosal, skin, and ocular involvement, in order to assess any potential correlation between these circulating biomarkers, disease activity, and the specific clinical features of disease. First of all, we found elevated levels of several inflammatory markers in BD patients including Chitinase3-like1, gp130/sIL-6Rb, IL-11, IL-26, sTNF-R1, and sTNF-R2 compared with HC. Comparing HC with disease activity, we observed enhanced levels of sTNF-R1 and sTNF-R2 in both active BD and inactive BD subgroups, while Chitinase3-like1 and gp130/sIL-6Rb serum levels were significantly higher in inactive BD as well as IL-26 in active BD than HC. Furthermore, M-BD patients showed enhanced levels of Chitinase3-like1, sTNF-R1, and sTNF-R2 compared with HC as well as increased levels of gp130/sIL-6Rb, sIL-6Ra, IL-11, IL-26, IL-35, sTNF-R1, sTNF-R2, and TSLP were found in MO-BD compared with HC. Interestingly, enhanced levels of gp130/sIL-6Rb, sIL-6Ra, IL-35, and sTNF-R1 between MO- and M-BD were also found.

Chitinase3-like1, an inflammatory biomarker of endothelial dysfunction without chitinase activity, is secreted from activated neutrophils and macrophages in several pathological conditions characterized by tissue injury and inflammation (30). In this regard, it has been demonstrated that Chitinase3-like1 correlates with the severity of skin lesions in patients with psoriatic arthritis but not in those with psoriasis vulgaris alone (31), being, however, its serum levels increased in psoriasis vulgaris and in generalized pustular psoriasis (32). Chitinase3-like1 has recently received considerable attention as marker for inflammation in BD patients (33, 34). Indeed a Korean study on 112 patients with BD whose main clinical features were recurrent oral ulcers and skin lesions, has shown increased serum levels of Chitinase3-like1 and a positive correlation with disease activity (17). These results are in agreement with Bilen et al. that showed higher serum levels of Chitinase3-like1 in BD patients compared with HC, albeit they did not correlate with disease activity (35). Similarly, we found enhanced levels of Chitinase3-like1 in M-BD group, even if its values were higher in the inactive BD group. Besides Chitinase3-like1, for the first time we observed the upregulation of IL-11 in our BD patients. This pleiotropic gp130-signaling cytokine, is able to induce anti-inflammatory and mucosal protective effects in a variety of animal models of acute and chronic inflammation, such as mucositis and inflammatory bowel diseases. In particular, IL-11 may exert anti-inflammatory effects by reducing cytokines production by macrophages (36). *In vitro* studies suggest that recombinant human IL-11 inhibits TNF-α, IL-1β, IL-12, IL-6, and nitric oxide production from activated macrophages reducing inflammation and tissue damage and promoting mucosal repair (37). Data from our study suggest that IL-11 does not correlate with disease activity and there are no significant differences between the active and inactive BD groups. Interestingly, we also found a higher level of IL-11 in the MO-BD group rather than in M-BD alone, even though it has been suggested that this cytokine is connected to repair processes of mucosal tissue damage (37).

Regarding gp130/sIL-6Rb, inactive BD showed higher values of this cytokine than HC. Gp130 also known as beta-subunit of the IL-6 receptor (sIL-6Rb) or CD130 is a ubiquitously expressed signal-transducing receptor that forms part of the receptor complex for several cytokines, including IL-6, IL-11, and IL-27 (38). Classically, IL-6 activates gp130 by binding a non-signaling cognate IL-6 receptor, which then leads to the initiation of JAK/STAT signaling, a pathway that is often constitutively switched on in several inflammatory processes (39). However, IL-6 responses can also be elicited through IL-6 trans-signaling mediated *via* a naturally occurring soluble IL-6R (40). Several biological processes, including the switch from neutrophil to mononuclear cell recruitment during inflammation, the leukocyte trafficking, activation, and apoptosis (41, 42), are due to IL-6 trans-signaling which is inhibited by a soluble form of gp130, in turn able to effectively bind the IL-6/sIL-6R complex and to prevent activation of membrane-bound gp130, modulating the severity of inflammatory responses (43, 44). The ability of soluble gp130 to downregulate the severity of inflammation and joint destruction in murine antigen induced arthritis has been demonstrated by a significant reduction in inflammatory infiltrate within the affected joints (45). Convincing proofs regarding the inflammatory role of the IL-6/sIL-6R complex derive also from the study of Curnow et al. aimed at proving an insufficient lymphocytes apoptosis in uveitis able to induce an inflammatory process through the trans-signaling pathway (46). In this regard, in our study, we found enhanced levels of gp130/sIL-6Rb, especially in MO-BD group than M-BD, although no correlation with disease activity was observed. Finally, a strong correlation between gp130/sIL-6Rb circulating levels and disease duration in MO-BD subgroup was also observed.

To the best of our knowledge, no studies have focused on the role of IL-26 in BD. In our study, serum concentration of IL-26 was significantly higher in BD, especially in active BD, than in HC. IL-26, a member of the IL-10 cytokine family, capable of inducing the production of several pro-inflammatory cytokines, such as IL-1β, IL-8 and TNF-α (16), is released in large amount in response to classic pro-inflammatory stimuli and enhances chemotaxis of neutrophils (47). Interestingly, this cytokine may impair the responsiveness to itself in certain structural cells such as colon epithelial cell line suggesting its pathogenic role in inflammatory bowel diseases. Indeed, increased infiltration of IL-26-positive Th17 cells was found in the colon of Crohn’s disease patients (48) and elevated expression of IL-26 mRNA was observed in the colon of pediatric-onset ulcerative colitis (49) as well as in tonsils and Payer’s patches in response to microbial stimuli, thus suggesting a pivotal role in mucosal immunity for this cytokine (50). Moreover, in some dermatological diseases, such as psoriasis, IL-26 has been found more highly expressed in lesions than in normal skin, proving an important function in regulating the innate immunity of epithelial cells (51). Despite this cytokine would seem more related to a mucocutaneous disease subset, in our experiment, IL-26 serum levels were found higher in MO-BD group than M-BD alone, consequently its increased values are not discriminating for the mucocutaneous involvement.

A reasonable explanation of this discrepancy may lie in the fact that IL-26 expression should be directly sought in the skin lesions rather than in the serum from BD patients since the major source of IL-26 is provided by infiltration of Th17 lymphocytes in inflamed tissue (52).

Tumor necrosis factor-α is the main cytokine involved in acute inflammatory responses and stimulates the release of other pro-inflammatory mediators. Endogenous mechanisms mediated through two distinct TNF receptor types 1 and 2 which are shed from cell surface as soluble forms (sTNF-R1 and sTNF-R2) may limit the systemic inflammation (53). As previously reported, increased serum concentrations of the soluble forms of membrane receptor for TNF-α, sTNF-R1, and sTNF-R2, have been found in active BD (54) and demonstrated in several rheumatic diseases (55–58), however, controversial are data regarding their putative role as markers of disease activity. In particular, Turan et al. reported that increased sTNF-R2 may serve as a marker of disease activity in BD, especially in those patients with arthritis, furthermore an increased TNF-R2 expression was found in mucosal and cutaneous ulcers where mast cells were identified as the major source for this receptor (21). On the contray, in line with our study, Düzgün et al. found significantly increased serum levels of sTNF-R1 in BD patients compared with HC, even though they did not reflect active disease (20). Interestingly, higher levels of sTNF-R were observed in our cohort of BD patients than in HC; however, no differences were observed between the patients with mucocutaneous plus ocular involvement and those with the sole mucocutaneous symptoms. More recently, Ke et al. have proven that sTNF-R1 released by skin-derived mesenchymal stem cells is critical for inhibiting the differentiation of Th17 cells, which are the major contributor of experimental murine models of autoimmune disease leading to IL-17A, IL-17F, IL-21, and IL-22 production (59). Similarly to what was observed for serum levels of gp130/sIL-6Rb, important results were obtained analyzing the values of sTNF-R1 and sTNF-R2 versus disease duration: in particular a positive strong correlation was found between sTNF-R1 circulating levels and disease duration in MO-BD subgroup, thus suggesting as previously reported that BD appears to have a less aggressive clinical course over time related to disease duration (60).

The main limitations of our study are represented by the sample size that did not allow us to ascertain the actual correlation of specific cytokines with the different subsets of disease as well as the absence of a disease control group. Moreover, at the time of samples collection, all of the patients were already taking immunosuppressive agents, which might have affected cytokine serum levels. Finally, we believe that not evaluating disease activity with outcome measures, due to the incompleteness of data, could represent another limitation of our study.

In conclusion, our findings were in agreement with several studies that showed that the immune response in BD is skewed toward a Th1 as well as Th17 pathway (61). Moreover, we observed the upregulation of chitinase3-like1 and IL-11 in BD. These molecules are produced by innate immune cells and lead to monocytes dendritic cell maturation and inhibition of TNF-α and IL-6 signaling (62, 63). Finally, this IL-6, TNF and Th17 signature could discriminate mucocutaneous patients with ocular involvement from mucocutaneous patients without ocular involvement, even if further studies in a larger cohort of patients as well as a comparison with disease group are necessary. Our preliminary data could contribute to improve the knowledge regarding the role of specific target for novel therapies or for a different and more suitable use of biologic drug currently available, suggesting a possible role of these cytokines in the induction of specific BD clinical manifestations.

## Author Contributions

GL (Giuseppe Lopalco), OL, and AL wrote the manuscript; LC, CF, and FI designed the study and finally revised the manuscript; OL, GL (Giovanni Lapadula), and CF performed the data analysis; VV, BF, MG, GL (Giuseppe Lopalco), LC took care of patients enrollment, follow-up of the patients, and data collection.

## Conflict of Interest Statement

The authors declare that the research was conducted in the absence of any commercial or financial relationships that could be construed as a potential conflict of interest. The reviewer GE and handling Editor declared their shared affiliation, and the handling Editor states that the process nevertheless met the standards of a fair and objective review.
